# An eye drop combination for treating *Staphylococcus aureus-induced* keratitis in rats: repurposing ibuprofen

**DOI:** 10.1038/s41598-026-48096-z

**Published:** 2026-04-24

**Authors:** Nevine L. Seiffein, Maher Abdel-Nabi Kamel, Ghada Hani Ali, Lamia Said Kandil, Amani Kazem, Sherien A. Abdelhady

**Affiliations:** 1 Department of Microbiology and Immunology, Faculty of Pharmacy, Pharos University in Alexandria, Canal El Mahmoudia Street, Beside Green Plaza Complex 21648, Alexandria, Egypt; 2 Department of Biochemistry, Medical Research Institute, Alexandria University, Alexandria, Egypt; 3 Department of Pharmacy, Alexandria Main Hospital, Alexandria, Egypt; 4 School of Pharmacy and Biomedical Sciences, University of Lancashire, Preston, UK; 5 Department of Pathology, Medical Research Institute, Alexandria University, Alexandria, Egypt; 6 Department of Pharmacology and Biochemistry, Faculty of Pharmacy, Horus University in Egypt, New Damietta, Egypt

**Keywords:** Bacterial keratitis, TLR4, IL-1β, MMPs, Levofloxacin, Ibuprofen, Diseases, Drug discovery, Medical research, Microbiology

## Abstract

Infectious keratitis is a life-hindering disease that has been one of the leading causes of blindness worldwide. The current study aimed to test a combination of levofloxacin and ibuprofen for treating Staphylococcus aureus-associated keratitis, with special emphasis on the potential antimicrobial and antibiofilm activities of the non-steroidal anti-inflammatory drug (NSAID) moiety. keratitis was induced in male Sprague Dawley rats by abrasion in the right eye after topical anesthesia, then the wounded eyes were inoculated with a 10 µl aliquot containing *Staphylococcus aureus*. Rats were divided into five groups: control, infected keratitis rats, infected keratitis rats treated with 0.5% levofloxacin, infected keratitis rats treated with 0.3% ibuprofen, and infected keratitis rats treated with 0.5% levofloxacin + 0.3% ibuprofen. Treatments were given as eye drops for two weeks. Then, the corneal tissues were isolated for investigations. The molecular study showed that, among the four groups, rats treated with combined eye therapy of 0.5% levofloxacin + 0.3% ibuprofen had the lowest expression levels of inflammatory mediators, metalloproteinases, corneal angiogenesis, and the proapoptotic BCL2-associated X (Bax). Histological investigation confirmed the transcriptional changes, with the rats that received combined eye therapy showing the best therapeutic outcomes.

**Supplementary Information:**

The online version contains supplementary material available at 10.1038/s41598-026-48096-z.

## Introduction

Bacterial Keratitis is one of the devastating ocular infections that might eventually lead to complete blindness. It is considered an emergency, requiring fast intervention^1^. Microbial keratitis accounts for 90% of all keratitis types, among which, bacterial keratitis represents 65 to 90% of all infectious keratitis. The degree of corneal damage correlated to infections depends initially on the virulence of the infectious agents and the state of the cornea^2^. However, it was also proven that geography and climate might affect the types of bacteria implicated in keratitis^3^. Among the Organisms that are usually implicated in infectious keratitis are: *Pseudomonas aeruginosa* (*P. aeruginosa*)^4^, coagulase-negative staphylococci, *Staphylococcus aureus* (*S. aureus*), and fungi, were the most often recovered bacteria from corneal scrapings^5^. In-depth insight into the infectious agents implicated in keratitis infections reveals that *S. aureus* is the world’s most significant cause of keratitis. *S. aureus* is considered to be the most virulent of all species of *Staphylococcus* and has a host-adhesion enhancement, evasion of the inborn immune system of humans, and a cytolytic effect on host cells, causing eye pain, photophobia, blurred vision, and ciliary injection^6^.

Contact lens wear, ocular surface illness, ocular trauma, and past ocular surgery were all common risk factors for keratitis^7^. A corneal epithelial defect with underlying stromal inflammation caused by reproducing bacteria characterizes infectious keratitis. The most common symptom is acute eye discomfort and redness^8^. In acute bacterial keratitis, neutrophils migrate and infiltrate the site of infection from the perilimbal circulation. This is associated with expression of inflammatory mediators such as interleukin-6 (IL-6), interleukin-1β (IL-1β), tumor necrosis factor-α (TNF-α), interleukin-8 (IL-8), intracellular adhesion molecule-1 (ICAM-1), and monocyte chemoattractant protein-1(MCP-1) and matrix metalloproteinases^9^. Furthermore, Toll-like receptor (TLR) members play an important role in recognizing pathogen-associated molecular patterns and are crucial components of innate immunity^10^.

Antibiotics remain the best treatment for bacterial keratitis, and a recent examination found that all common topical antibiotics are equally effective among topical fluoroquinolones. Systemic quinolones such as ciprofloxacin, moxifloxacin, and levofloxacin are relatively good at penetrating the vitreous, but they may not exceed all possible pathogens’ minimum inhibitory concentrations (MICs). Ciprofloxacin, moxifloxacin, and levofloxacin have been accepted as monotherapies by the US Food and Drug Administration for Bacterial Keratitis treatment^6^. Some corneal specialists added corticosteroids to reduce bacterial keratitis scarring for their potent antiinflammatory effect; however, they suppress the immune system, do not affect acuity or scar size, and their re-epithelialization was considerably delayed^11^. Corticosteroids may still be associated with less favorable results in keratitis related to certain micro-organisms, such as Nocardia and atypical Mycobacteria, even with antimicrobial agents^12^. Such controversy prompted the use of local non-steroidal anti-inflammatory drugs (NSAIDs) for treating bacterial keratitis, which have the same anti-inflammatory effects as steroids and the added benefit of lowering the recurrence rate^13^. NSAIDs often provide quick analgesic effects for the cornea lasting up to 24 h, and are generally more effective than topical corticosteroids for relieving ocular pain^14^. Therefore, NSAIDs are a valuable alternative to steroid therapy in treating ophthalmic conditions, which are easy to use, have a high patient compliance rate, are more cost-effective than surgery for treating corneal opacity, and enhance visual acuity^13^. However, topical NSAIDs cause corneal melting, a rare and most severe sight-threatening effect, by preventing the synthesis of prostaglandin E2 (PGE2), which has a protective influence on corneal integrity^15^. Also, preservatives (mainly benzalkonium chloride) in topical medications exert a toxic effect upon corneal epithelium^16^. Some topical NSAIDs with antiseptic, lubricants, and moisturizing properties are also used preoperatively in cataract surgery to improve the ocular surface and potentially to reduce the risk of postoperative infection^16^.

Investigating the effect of different treatments on the initiation of inflammation initiation^17^, proliferation^18^, and apoptosis^19^ in corneal epithelial cells is the basis for the evaluation of the therapeutic efficacy of chosen treatments. Hence, this study aimed to (i) detect the effect of ibuprofen, 0.5% levofloxacin, and ibuprofen + 0.5% levofloxacin combined eye drops against ocular Staphylococcus isolates, (ii) test the efficacy of the chosen drugs on (S. aureus)-induced keratitis of rats, and (iii) design a new formulation containing a combination of both ibuprofen and levofloxacin.

## Results

### Identification

*S. aureus* isolates were obtained from laboratories and were further identified by growth on mannitol salt agar. All those that were included in the study were both cefoxitin and oxacillin-resistant depicting MRSA, all isolates were catalase-positive, 80% coagulase-positive and 65% B hemolytic.

### Kirby Bauer results

The levels of resistance among The twenty-five *S. aureus* isolates under test after interpretation of zone diameters obtained by Kirby Bauer test were: amikacin 100% amoxiclav 85%, ampicillin 90%, ampicillin/sulbactam 85%, azithromycin 20%, cefadroxil, cefipime, cefoperazone, cefoperazone/sulbactam, cefotaxime ceftazidime, ceftriaxone, cefuroxime, ertapenem, meropenem and imipenem 87%, ciprofloxacin 40%, levofloxacin 23% moxifloxacin 18%, ofloxacin 25%, clindamycin 10%, doxycycline 30%, erythromycin 50%, fusidic acid 50%, gentamicin 76%, sulfamethoxazole/trimethoprim 78%, oxacillin 100%, cefoxitin 100% and completely sensitive to tigecycline, linezolid and vancomycin. levels of resistance are expressed as percentage prevalence of the antibiotic resistance among the 25 *Staphylococcus aureus and it was interpreted using CLSI M100 reference*.

*CLSI. Performance Standards for Antimicrobial Susceptibility Testing. 35th ed. CLSI supplement M100. Clinical and Laboratory Standards Institute; 2025.*

### Determination of biofilm-forming capacity of all isolates

**Fig. 1:**
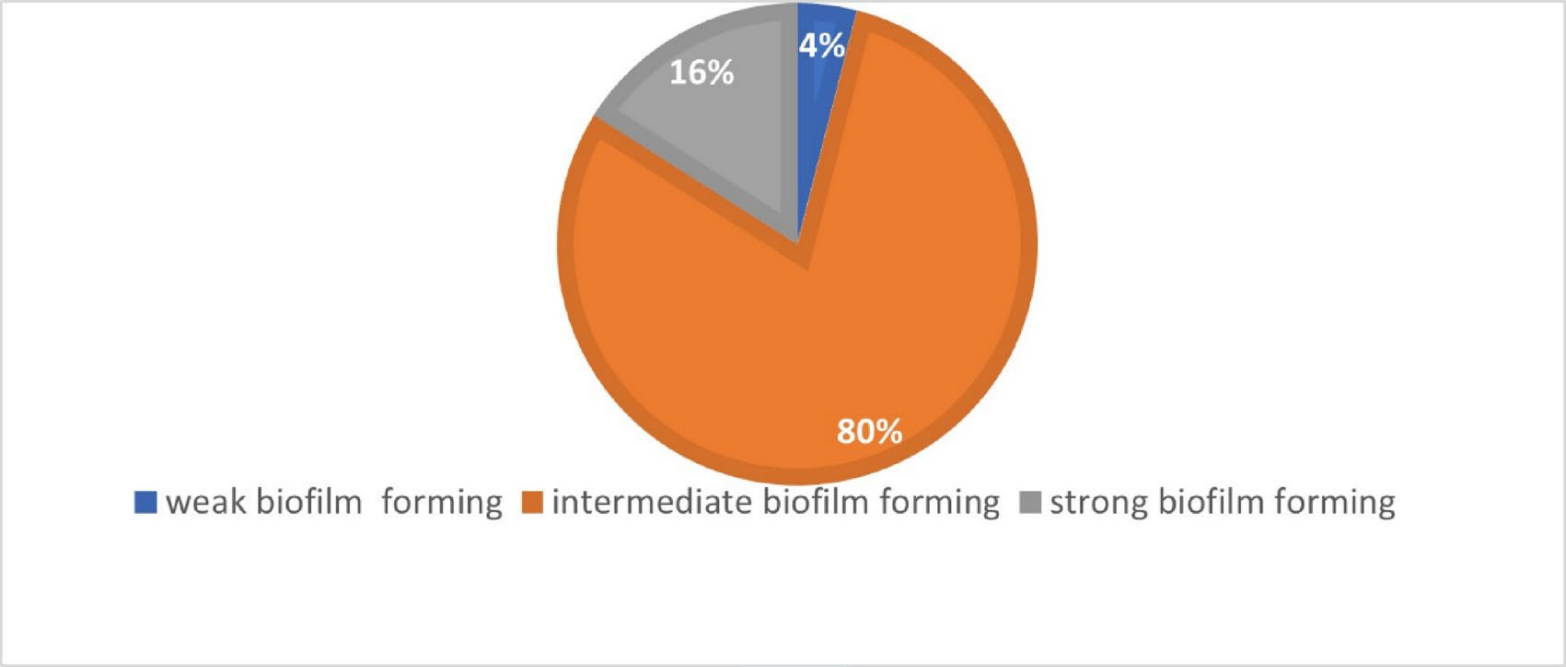
Biofilm forming capacities among *Staphylococcus aureu*s isolates under test.

The biofilm-forming capacities of the *S. aureus* isolates under test 16% were strong biofilm formers as shown in Fig. 1.

### Detection of antimicrobial activity of Ibuprofen and its effect on levofloxacin

The checkerboard arrangement of the experiment showed that the MIC of levofloxacin against the 20 *S. aureus* isolates ranged between 3.9 and 7.8 ug/ml. The MIC of ibuprofen was 46.8 ug/ml.

The combination of ibuprofen with levofloxacin led to a decrease in its MIC till it reached less than 0.9 ug/ml i.e. a decrease in MIC by 6 to 8-fold such result denotes synergism, such behavior was noted for all isolates.

### Determination of the antibiofilm activity of ibuprofen alone and in combination with levofloxacin antibiotic

**Fig. 2:**
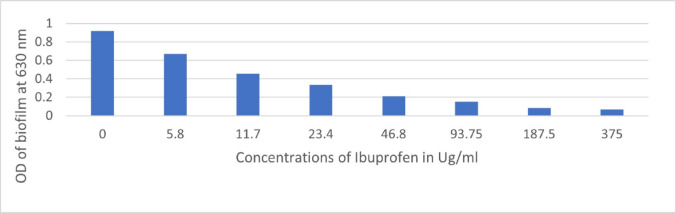
Decrease in biofilm mass by increasing ibuprofen concentration.

Figure 2 shows that the ibuprofen alone at increasing concentrations between 5.8 ug/ml and 375ug/ml decrease greatly and significantly the formed biofilm mass by *S. aureus.* Calculated as follows Biofilm mass in presence of ibuprofen-biofilm mass in absence of ibuprofen/the biofilm mass in absence of ibuprofen.

### Effect of increasing concentrations of ibuprofen on ability of levofloxacin antibiotic to prevent biofilm formation

Combining the different concentrations of levofloxacin with different concentrations of ibuprofen affected biofilm mass as the presence of the anti-inflammatory drug slightly assisted the biofilm prevention activity of levofloxacin a 90% decrease in biofilm mass, which is highly significant as almost no biofilm was formed calculated as follows.

**Fig. 3:**
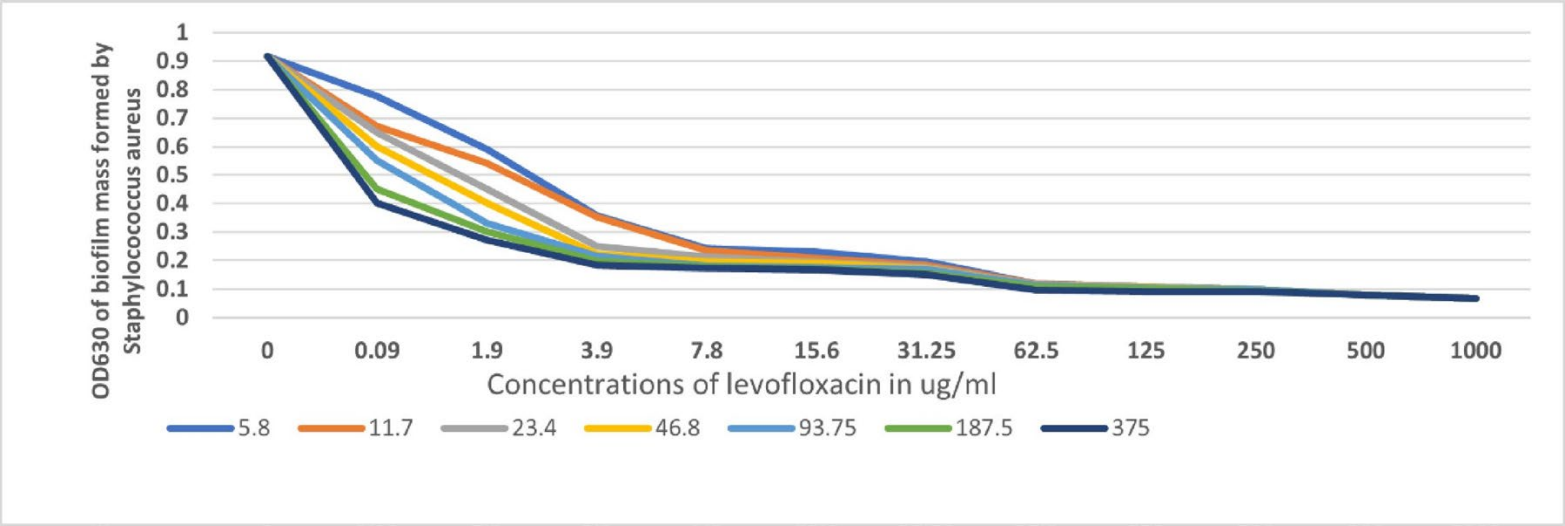
Effect of increasing concentrations of ibuprofen on the ability of levofloxacin antibiotic to prevent biofilm formation.

Biofilm mass in absence of ibuprofen (combined with levofloxacin) -biofilm mass in its absence/Biofilm mass in its absence (Fig. 3).

### The determination of MIC of the prepared formulations

When the MICs of the three formulations were determined in vitro using the broth microdilution assay, the results were as follows:

Plain formulation (negative Control): Completely inactive against *S. aureus* isolates, where no killing was observed under test conditions (as seen by turbidity in all wells).

Formulation with 0.5% levofloxacin MIC ranged between 7.8 and 15.6 ug/ml.

Formulation with 0.3% ibuprofen MIC = 18.75 ug/ml.

Combination formulation MICs at concentration 2.34 ug/ml for ibuprofen and 3.9 ug/ml for levofloxacin.

### The prevention of biofilm formation by the three formulations under test

As we can deduct from Fig. 3, there was a decrease in the biofilm-formed mass by the three formulations, with the highest effect attributed to the combination formulation.

###  The effect of the three formulations on the bacterial count

**Fig. 4:**
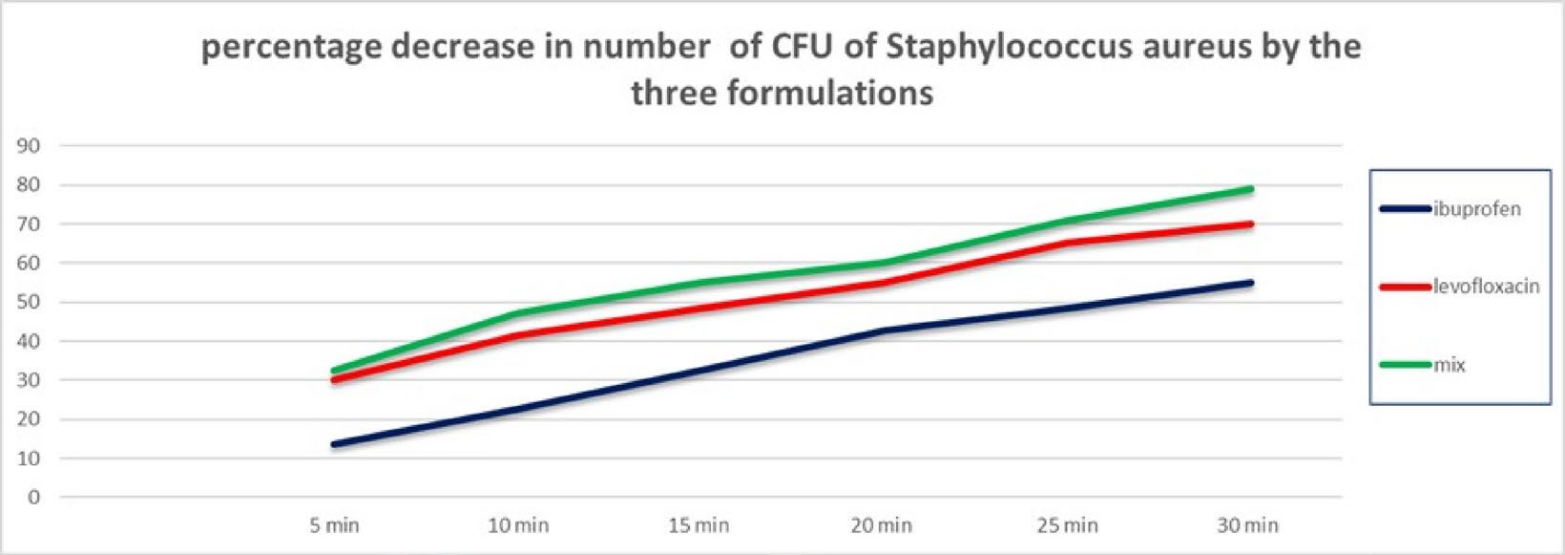
Percentage decrease in the number of CFU of *S. aureus* by the three formulations under test.

Using a starting bacterial count of 8.9 × 10^5^ CFU/ml as it appears in Fig. 4, where there was an increase in the percentage decrease in the count of bacteria under test (Fig. 4).

### Prevalence of tested genes among the *S. aureus* isolates

PCR detection revealed the prevalence of genes among *S. aureus* isolates (Table 1).

**Table 1:** Genes prevalence among *S.* aureus isolates.

gene	Prevalence in 25 isolates
**mecA** ^**1**^	100%
**Ica** ^**2**^	100%
**hla**	88%
**sirB**	92%
**ebpS**	84%
**Pvl**	68%
**Tsst**	64%
**Sea**	56%
**sdrE**	64%
**Cna**	36%
**fnbA**	56%
**fnbB**	12%
**clfA**	20%
**Hlb** ^**3**^	12%
**Hlg** ^**4**^	4%
**Eta** ^**5**^	0%
**scpA** ^**6**^	0%
**LUKdE** ^**7**^	0%

MecA^1^, one of the genes coding for methicillin resistance.

Ica^2^, gene is involved in the synthesis of poly N-acetylglucosamine for intercellular adhesion;.

Hlb^3^ and Hlg^4^, Hemolysin genes.

Eta^5^ gene, encodes a member of the Y family of specialized DNA polymerases. It copies undamaged DNA with a lower fidelity.

scpA^6^, staphopain.

ALUKDE^7^, leucocidin genes.

### Molecular docking pose

**Fig. 5:**
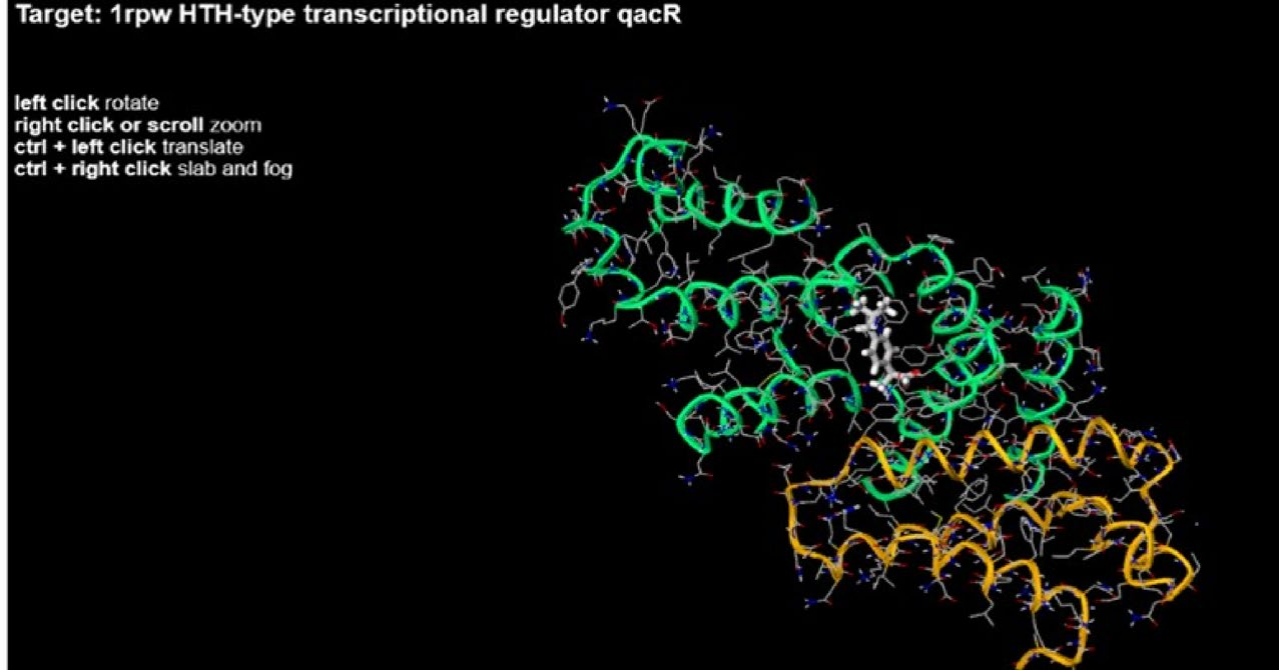
Pose of ibuprofen against HTH-type transcriptional regulator.

When the structure of Ibuprofen was tested against different targets on the Mcule, the pose in Fig. 5 was of −8 magnitude with helix-turn-helix (HTH) type transcriptional regulator qacR. annotated as a putative HTH type transcriptional regulator and QacR, is the multidrug export protein whose expression it regulates, has been shown to interact directly with many structurally dissimilar compounds (Fig. 5).

### Effect of drug therapies on corneal inflammation

**Fig. 6:**
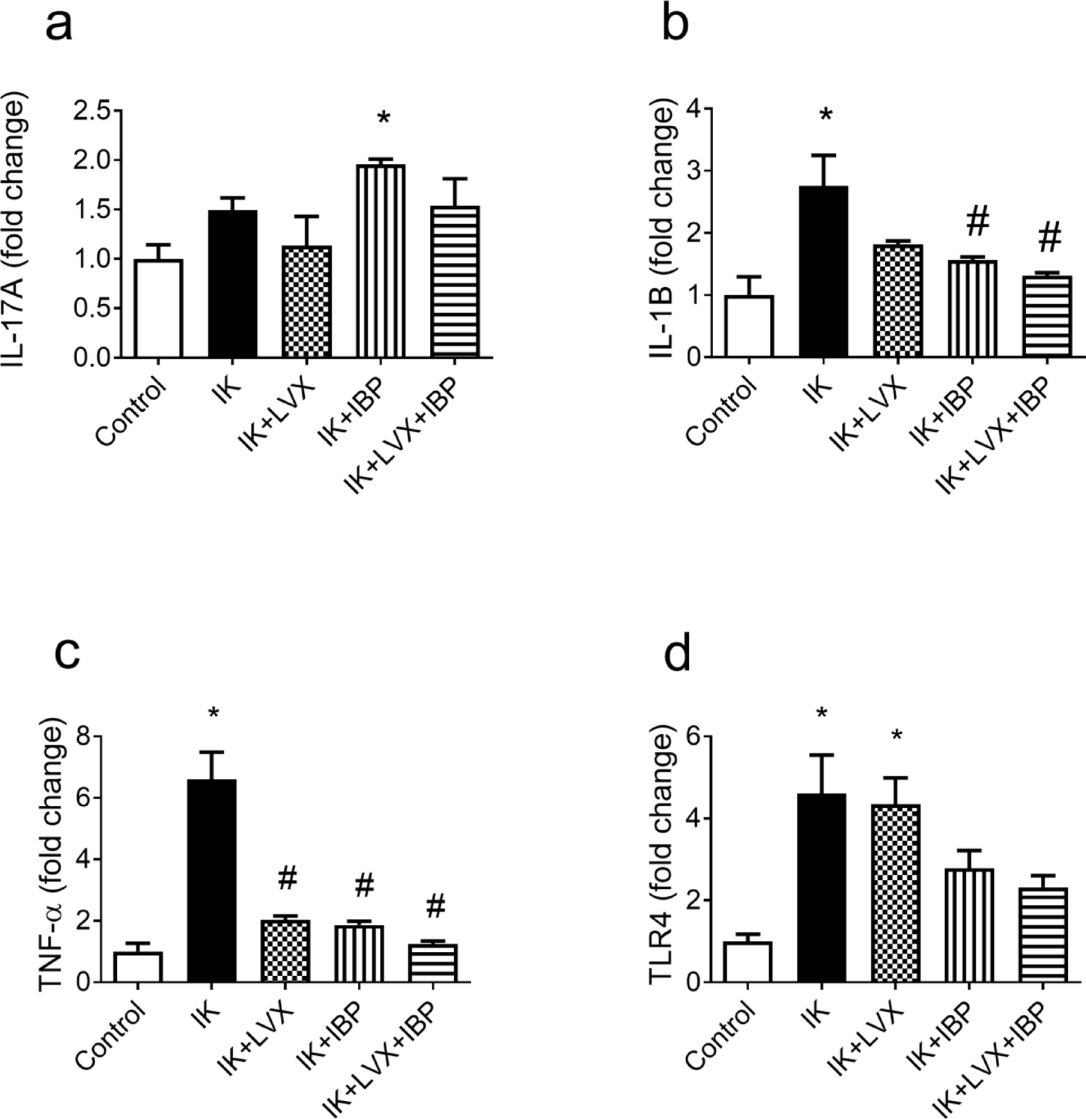
**Effect of drug therapies on gene expression of inflammatory mediators**. Interleukin 17A (IL-17 A, panel a), interleukin-1β (IL-1β, panel b), tumor necrosis factor-α (TNF-α, panel c), toll-like receptor 4 (TLR4, panel d). Data are presented as means ± SD (n=3). Comparisons between groups were analyzed using one-way ANOVA followed by Tukey multiple comparison test. Data are compared with respective keratitis (*) and IK (#). at p<0.05. IK, infected keratitis; LVX, levofloxacin; IBP, ibuprofen.

RT-PCR studies displayed that, compared with the control group, significant increases in gene expression of IL-1β (*p* = 0.0065), TNF-α (p˂0.0001), and TLR4 (*p* = 0.006) were shown in the infected keratitis rats’ group (Fig. 6B-D). However, no significant change was observed in IL-17 A expression in the cornea of the infected keratitis group (Fig. 6A). The upregulation of IL-1β expression was significantly decreased by treatment with ibuprofen (p˂0.05) and the combined therapy (levofloxacin+ibuprofen) (p˂0.05) (Fig. 6B). Elevated gene expression of TNF-α was decreased significantly by all the treated groups (levofloxacin group; *p* = 0.0001, ibuprofen group, *p* = 0.0001; and levofloxacin+ibuprofen group, p˂0.0001) (Fig. 6C). The expression levels of TLR4 mRNA were significantly increased in the infected keratitis rats (*p* = 0.006) and levofloxacin-treated groups (p˂0.05) compared to control rats, while there were no significant changes observed in TLR4 expression in the groups treated with ibuprofen and/or combined therapy of levofloxacin+ibuprofen when compared with the control rats (Fig. 6D).

**Fig. 7:**
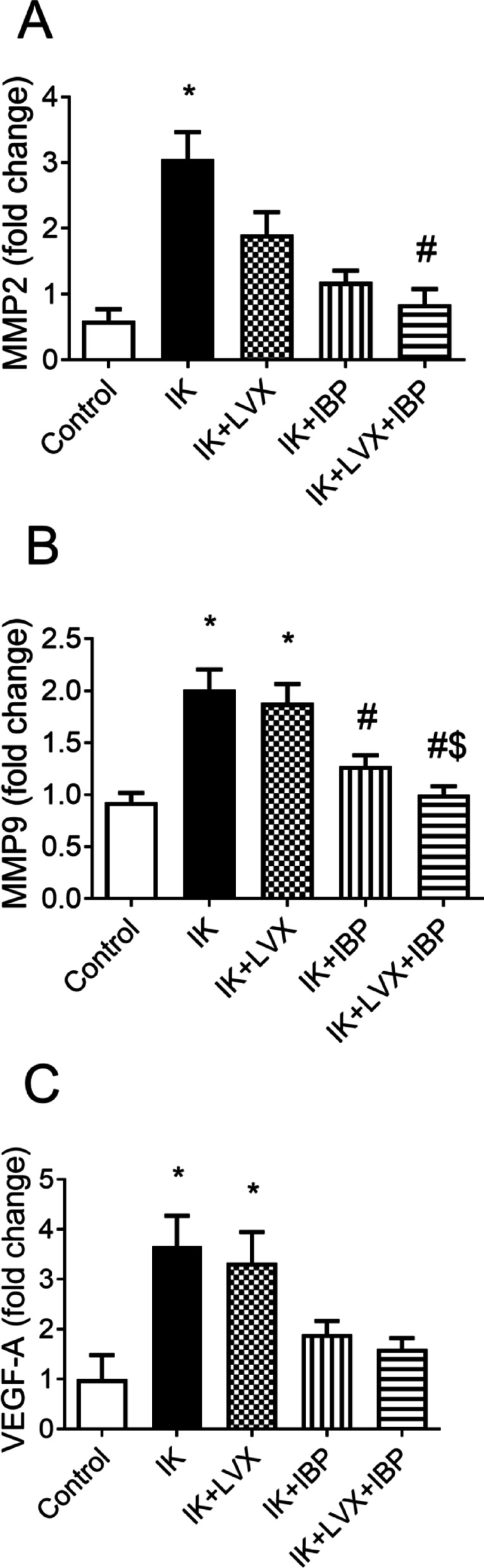
Effect of drug therapies on gene expression of metalloproteinases, matrix metalloproteinase-2 (MMP2, panel a), matrix metallopeptidase 9 (MMP9, panel b), and gene expression of Vascular endothelial growth factor A (VEGF-A, panel c). Data are presented as means ± SD (*n* = 3). Comparisons between groups were analyzed using one-way ANOVA followed by Tukey multiple comparison test. Data are compared with respective keratitis (*), IK (#), and LVX ($). at *p* < 0.05. IK, infected keratitis; LVX, levofloxacin; IBP, ibuprofen.

The mRNA expression of both MMP-2 and MMP-9 in the infected keratitis group showed significant up-regulation compared to the control cornea rats (*p* = 0.0172, *p* = 0.0005, respectively). Treatment with levofloxacin+ibuprofen therapy revealed significant (*p* = 0.0172) downregulation of MMP-2 expression compared with the untreated infected keratitis group (Fig. 7A). The maximum fold change in MMP-9 expression was observed in the levofloxacin-treated infected keratitis group, with no significant changes when compared with the infected keratitis rats. However, rats treated with ibuprofen (p˂0.05), and/or combined therapy (levofloxacin+ibuprofen, *p* = 0.0005) showed significant downregulation of MMP-9 expression compared with the untreated bacterial keratitis rats. Moreover, fold change in MMP-9 expression substantially decreased in rats treated with levofloxacin+ibuprofen biotherapy compared with the levofloxacin group, *p* = 0.0005 (Fig. 7B).

VEGF-A expression levels were significantly higher in both the untreated bacterial keratitis group and the levofloxacin-treated rats (*p* = 0.0108) than in the control group; nonetheless, there were no differences between rat groups treated with ibuprofen and/or combination therapy and the control groups (Fig. 7C).

**Fig. 8:**
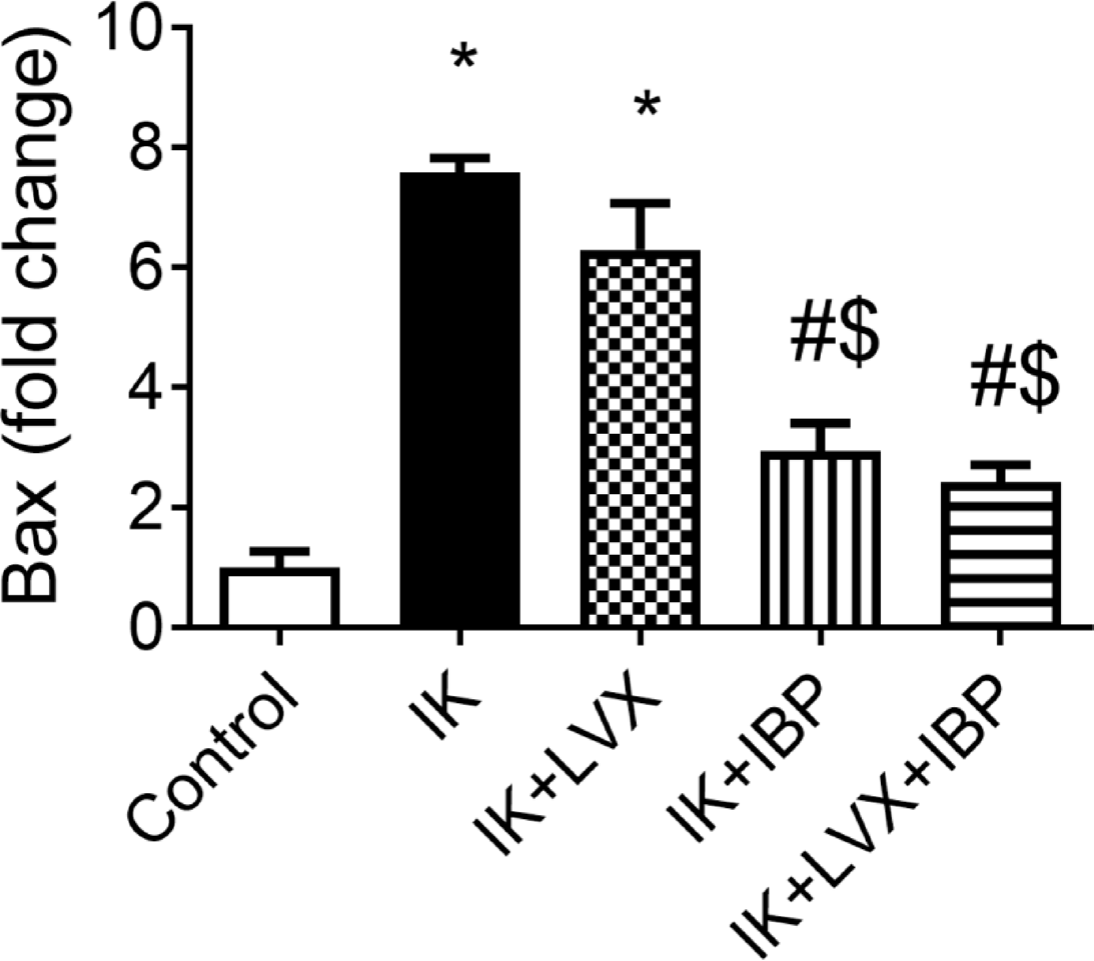
Effect of drug therapies on gene expression of apoptotic marker (Bax). Data are presented as means ± SD (*n* = 3). Comparisons between groups were analyzed using one-way ANOVA followed by Tukey multiple comparison test. Data are compared with respective keratitis (*), IK (#) and LVX ($). at *p* < 0.05. IK, infected keratitis; LVX, levofloxacin; IBP, ibuprofen.

The expression level of Bax mRNA was significantly increased in the untreated infected keratitis rats (p˂0.0001) and the levofloxacin group (p˂0.0001) compared with the control rats. This abnormal upregulation was substantially lowered after ibuprofen (p˂0.05) and/or levofloxacin+ibuprofen (p˂0.0001) biotherapy treatments compared to bacterial keratitis and levofloxacin groups (Fig. 8).

### Effect of drug therapies on corneal structure

**Fig. 9:**
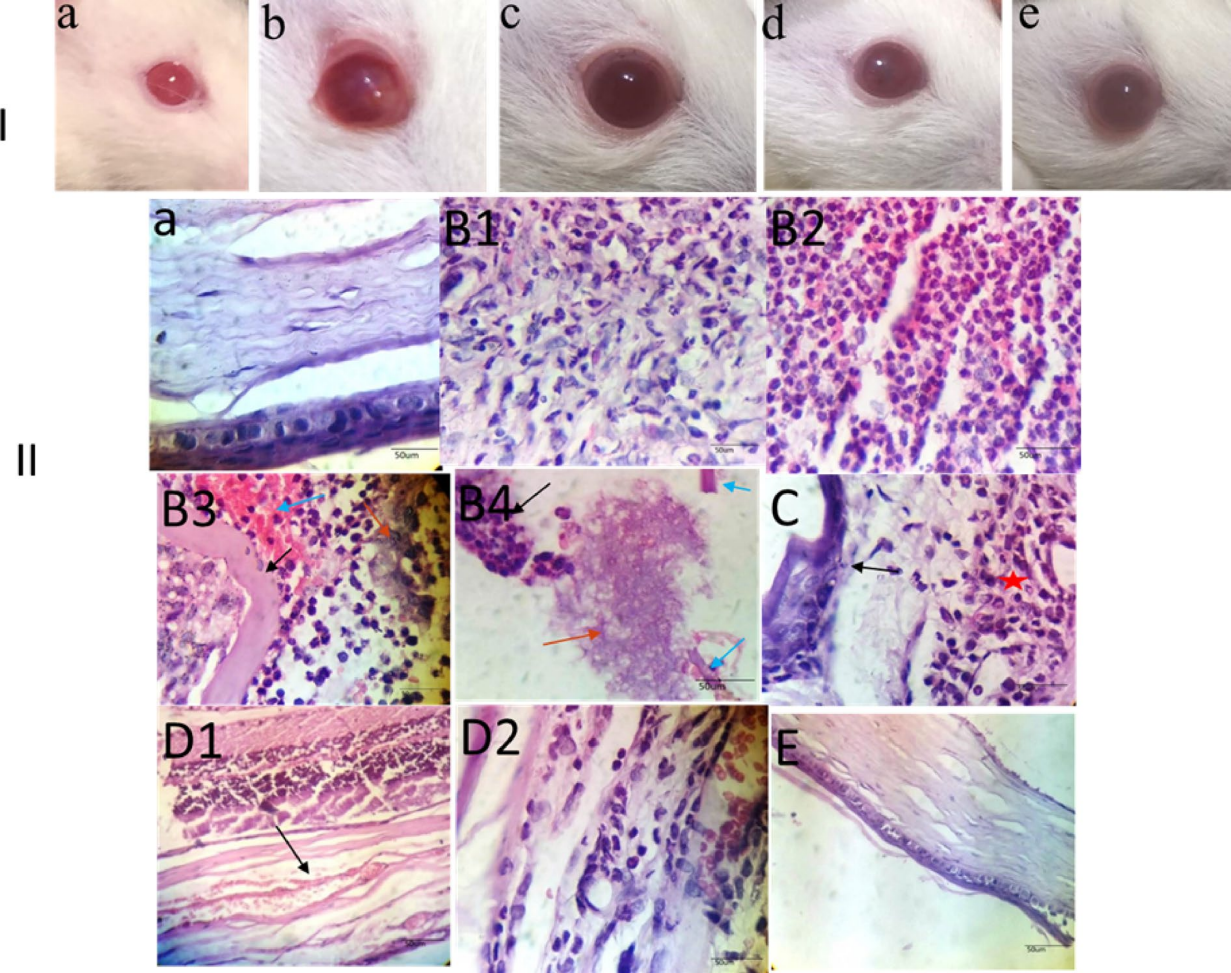
**Panel I: An illustrative shoot of the studied animal groups;** (a) Control, (b) Infected keratitis, (c) LVX, (d) IBP, (e) LVX + IBP. **Panel II**: **H&E staining of rats’ cornea sections (400x)**. Normal cornea **(a)**. Infected keratitis in rats illustrating acanthotic corneal epithelium heavily infiltrated by polymorphonuclear leukocytes **(b1)**, acute corneal abscess **(b2)**, detachment of corneal epithelium from the basement membrane by suppurative inflammatory infiltrate and hemorrhage (blue arrow), separated epithelium (red arrow) and thickened basement membrane black arrow) **(b3)**, bacterial colonies (red arrows) surrounded by polynuclear leukocytes (black arrows) with fragmented basement membrane (blue arrow) **(b4)**. Levofloxacin treated group revealing irregular thickening of the corneal epithelium (arrow) with underneath vascularized stroma moderately infiltrated by acute inflammatory cells (star) **(c)**. Ibuprofen treated group showed neovascularization (arrow) **(d1)** as well as acute and chronic mononuclear inflammatory infiltrate **(d2)**. Levofloxacin+ibuprofen group proving near normal architecture of cornea with absent inflammation **(e)**.

Figure 9 revealed that the untreated bacterial keratitis rats showed acanthotic corneal epithelium heavily infiltrated by inflammatory cells with hemorrhage and separated epithelium. Both levofloxacin and ibuprofen-treated groups revealed the presence of moderate inflammation and neovascularization. However, the rats group treated with the combined therapies of levofloxacin+ibuprofen demonstrated limited corneal abnormalities.

## Discussion

Infectious keratitis is more prone to cause corneal lesions than noninfectious keratitis^6^. Bacterial keratitis is regarded as the most common form of infectious keratitis, which is responsible for fibrosis in the central visual axis, reducing visual acuity, and corneal transplantation is recommended in severe cases^20^. In particular, *S. aureus* is the most implicated organism, which is capable of developing antibiotic resistance, making this infection one of the most difficult infections to treat^21^. In the present study, the *S. aureus* isolates were detected to be multidrug-resistant, as this behavior is responsible for the failure of antibiotic treatment, the urge to a combination of more than one antimicrobial agent or an antibiotic and a repurposed nonantibiotic evolved is in concordance with the research of Egrilmez, S et al.^22^ where they suggested the use of combination therapy to overcome the emerging resistant organisms. In addition, it was shown that all the isolates under test were biofilm formers with the highest percentage (89%) of intermediate capacity, researchers^23^ detected a high ability of biofilm formation among the *S.aureus* keratitis isolates such ability is one of the key factors underlying the virulence of the microorganisms and their pathogenicity. Therefore, it might lead to vision loss putting into consideration that biofilm-forming microorganisms are more virulent than non-biofilm formers, as well the organisms were 100% methicillin-resistant, 100% harbored the ica gene which correlates perfectly with the ability of all the isolates to produce biofilm as it was stated by Thilakavathy et al.^24^ that the operon is essential for the adhesion ability, in addition, etA, scpA, and LUKD/E were absent with a low prevalence of Hlg and HlB.

The levofloxacin minimum inhibitory concentration was determined among the *S. aureus* isolates, and it ranged between 3.9 and 7.8 ug/ml. The MIC of ibuprofen was 46.8 ug/ml; other researchers, such as El-Janabi et al. reported a MIC of 1.25 mg/ml for ibuprofen and acetaminophen against *S. aureus*, such variation can be attributed to the variation in the phenotypic and genotypic profile of isolates in Egypt^25^. Ibuprofen was chosen due to its high safety profile and multiple mechanisms, and its use in keratitis is accepted to decrease inflammation due to its activity as a COX inhibitor^26^. Ibuprofen is known to show antimicrobial activity towards many microorganisms, including the mycobacterium tuberculosis^27^ and cystic fibrosis-associated pathogens^28^. The activity of the levofloxacin was also tested in vitro alone and in combination with ibuprofen showed a decrease in the MIC of the levofloxacin, noted as synergism, the same effect was noted for the formulation in vitro. The same effect was noted^29^ with salicylates in the form of a decrease in antibiotic resistance by certain bacteria exposed to salicylates and reported that ibuprofen is associated with an increase in the efflux of antibiotics via the AcrAB-TolC efflux pump in a manner dependent on the transcriptional regulator MarA. Moreover, it was detected^30^. Ibuprofen in particular induces lower antibiotic resistance compared to other NSAIDs and shows complete MarA dependence.

In the present study, in an attempt to elucidate the mechanism by which ibuprofen performs its action, further investigation through docking using Mcule free software was done. It suggests a potential interaction between ibuprofen and the HTH-type instructional regulator associated with multidrug resistance. That may support both the activity and the synergistic effect of ibuprofen with levofloxacin. Researchers attributed the antimicrobial activity of ibuprofen, a non-selective COX inhibitor, to an increase in superoxide and hydroxyl radicals that cause DNA strand scission^31^. Other researchers as Abbas et al.^32^ attributed the antimicrobial activity of ibuprofen in *S. aureus *to blocking AgrA-regulated virulence, inhibiting hemolysis, and hindering staphyloxanthin production. On the other hand, some researchers found that ibuprofen was implicated in the induction of antibiotic resistance^33^. In our study, ibuprofen greatly decreased the biofilm-formed mass either alone or in combination with levofloxacin, it was demonstrated positive results regarding the anti-biofilm formation of ibuprofen against *P. aeruginosa*^34^. Furthermore, they found a concentration-dependent reduction in biofilm formation with rising drug concentrations (0, 50, 75, and 100 µg/mL). Concerning *S. aureus*, it was detected by Abbas et al.^32^ that ibuprofen downregulates the expression fnbA and icaA genes, which are crucial in biofilm formation.

In infectious keratitis, activated corneal cells or corneal fibroblasts recognize bacterial constituents *via* TLR family members expressed on the ocular surface^35^. TLR4 is one of the ultimate important receptors in the innate ocular immune system through which infectious and non-infectious attacks result in inflammation^36^. In the current study, a model of induced bacterial keratitis was designed, and the results showed increased inflammation in both infected keratitis and levofloxacin-treated rat groups, as evidenced by increased corneal expression of TLR4 that was downregulated in groups treated with ibuprofen and/or levofloxacin+ibuprofen. These rats, who received levofloxacin+ibuprofen eye therapy, also showed the lowest expression levels of corneal IL-1β, a proinflammatory cytokine produced by ocular mucosal epithelial cells, whose primary function is to upregulate inflammation^37^. These findings suggest modulation of inflammatory signaling by treatment with the levofloxacin+ibuprofen combined therapy. Likewise, Hsu et al. reported that the infectious keratitis and levofloxacin groups had high levels of IL-1β and TNF-α expression^38^. Corneal epithelial cells provide the first line of host defense against pathogens, recognize invading pathogens, and can trigger the secretion of cytokines that recruit inflammatory cells to kill the pathogens^39^. Rats infected with bacterial keratitis have approximately the same levels of IL-17 A expression, a characteristic protein of Th17 inflammatory signaling^40^, as the keratitis rats; however, ibuprofen-treated rats showed significant upregulation of corneal IL-17 A. Our results are consistent with those reported by Arranz-Valsero et al., who showed no upsurge in IL-17 A after stimulation with bacterial supernatant in human corneal epithelial cultures, denoting that corneal epithelial cells may not be the major source of IL-17 during infectious inflammation^40^.

The penetration of the pathogen induces the expression of various chemokines and adhesion molecules in corneal fibroblasts^35^ and promotes local infiltration and activation of neutrophils, which contribute to the clearance of the pathogen^41^. However, the corneal fibroblasts activated by factors released by bacteria are essentially responsible for making collagenolytic MMPs for the destruction of extracellular collagen correlated with corneal ulcers^42^. Furthermore, it was found that IL-1β stimulated the production of MMP1, MMP3, and MMP9 by corneal fibroblasts cultured in collagen gel and increased collagen degradation^43^. This finding agrees with our results, with the bacterial keratitis group showing elevated levels of IL-1β, MMP2, and MMP9. Metalloproteinases (MMPs) were significantly downregulated by levofloxacin/ibuprofen combination therapy, which may be concomitant with diminished proteolytic signaling pathways. While combination therapy significantly reduced MMP-2 and MMP-9 mRNA expression, these findings reflect transcriptional regulation and do not directly confirm protein level changes. Future studies using protein-level measurements and functional assays such as gelatin zymography will be necessary to confirm these effects. MMPs have been reported as potential therapeutic targets to prevent collagen degradation by corneal fibroblasts^42^. Given that corneal fibroblasts are the source of MMPs involved in stromal collagen dissolving, targeting these cells instead of MMPs themselves is a prospective alternative therapeutic attempt for corneal ulcers^44^. However, within the 14-day treatment, the combined therapy did not establish histological evidence of corneal melting.

The cornea of ​​the eye is transparent and lacks blood vessels in a healthy state^45^. Keratitis often causes VEGF-dependent new blood vessel growth, and stimulation of corneal limbal fibroblasts by *S. aureus * induces VEGF-A, which can lead to abnormal blood vessel formation in the normally avascular cornea^46^. Additionally, corneal epithelial cells produce VEGF through TLRs^47^. Neovascularization is a common obstacle to healing in corneal infections, resulting in permanent inflammation, edema, lipid buildup, tissue scarring, and reduced corneal clarity and vision^48^. Therefore, controlling the level of corneal neovascularization in bacterial infectious keratitis is essential, along with bacterial suppression^49^. The current study showed high expression of VEGF-A mRNA in both the bacterial keratitis and levofloxacin-treated rat groups, which also exhibited higher TLR4 expression than other groups. VEGF-A upregulation was most significantly reduced by combination therapy with levofloxacin and ibuprofen. Histological examination confirmed these molecular findings: the levofloxacin-treated group displayed angiogenic stroma with moderate infiltration of acute inflammatory cells, the ibuprofen-treated group showed neovascularization, and the combined levofloxacin/ibuprofen group had corneal structures close to normal.

Bax has been reported to increase mitochondrial outer membrane permeability, leading to the release of mitochondrial apoptotic components such as cytochrome c, apoptosis-inducing factor, and endonuclease G, which translocate to the nucleus and induce significant chromatin condensation and/or DNA fragmentation^50^. Proapoptotic Bax was upregulated in the bacterial keratitis group and downregulated after treatment with the combined levofloxacin and ibuprofen therapy, which proposes alteration of apoptotic signaling pathways. Histological examination strengthened the biological relevance of the observed molecular changes, revealed a clearly reduced inflammatory infiltrate and amended tissue organization compared with untreated infected corneas after 14 days of treatment with the levofloxacin+ibuprofen combined therapy. Although topical NSAIDs have been associated with corneal epithelial complications, our histological findings did not reveal structural damage or epithelial loss in the combination therapy group. However, direct assessment of epithelial integrity using fluorescein staining or corneal thickness measurements was not performed and should be included in future safety evaluations. At all, corneal epithelial cells of the bacterial keratitis, levofloxacin, and/or ibuprofen groups showed detachment of the corneal epithelium from the basement membrane due to acute corneal abscess and inflammatory infiltration. Therefore, treatment with levofloxacin+ibuprofen was associated with improved transcriptional and histological markers, suggesting potential therapeutic benefit in a preclinical rat model that requires further validation.

Conclusion: Our study identifies transcriptional changes in the rat cornea associated with *S. aureus* keratitis and their modulation after treatment with ibuprofen, levofloxacin, or a combination of both. These abnormal mRNA expressions in inflammatory cytokines, matrix metalloproteinases, and angiogenic factors suggest potential pathways involved in the corneal response to infection and treatment. However, the current study displays exploratory rather than confirmatory findings due to several limitations that should be addressed in future research. These include the short treatment duration of 2 weeks, as longer periods may be necessary to fully assess efficacy and safety; the limited evaluation of potential adverse effects of the combination therapy such as corneal epithelial integrity and implementation of blinded histological scoring; the focus on only *S. aureus* without examining other common keratitis-causing pathogens like *Pseudomonas aeruginosa*; the use of fixed concentrations of levofloxacin and ibuprofen without dose-ranging studies for optimization; a small in vivo sample size; reliance on transcriptional data without assessing protein levels; limited statistical power; only a few potential molecular mechanisms explored; and the need for a more comprehensive analysis of how ibuprofen enhances levofloxacin’s effects. This study provides preclinical evidence supporting the potential synergistic anti-inflammatory and antimicrobial activity of combined levofloxacin and ibuprofen. However, further studies with larger sample sizes, dose-response analyses, and protein-level mechanistic validation are necessary before clinical translation.

## Materials and methods

### Materials

Levofloxacin HCL powder was generously provided by PHARAONIA PHARMACEUTICALS (Cairo, Egypt). Ibuprofen was obtained from Kahira Pharmaceuticals and Chemical Industries Co (Cairo, Egypt). Glacial acetic, crystal violet, and methanol were purchased from Elgomhoreya company Egypt. Antibiotic discs and media were obtained from Oxoid.inc Catalog numbers as follows: Mannitol salt agar-(CM0085B), TSB agar-(CM1065B), Blood agar-(CM0055B), Antibiotic discs as follows: Oxacillin 1ug-(CT0159B), Cefoxitin 30ug-(CT0119B), Amikacin 30ug-(CT0107B), Amoxiclav-(CT0223B), Ampicillin 25 ug- (CT0004B), Ampicillin/sulbactam-(CT0520B), Azithromycin 15 ug-(CT0906B), Cefadroxil 30ug-(CT0453B), Cefepime 30ug-(CT0771B), Cefoperazone- (CT0249B), Cefoperazone/sulbactam 105 ug-(CT1727B), Ceftazidime 30ug-(CT0412B), Ceftriaxone 30 ug-(CT0417B) Cefuroxime 30ug-(CT0127B) Ciprofloxacin 5ug –(CT0425B), Clindamycin 2ug-(CT0064B), Doxycycline 30ug -(CT0018B), Ertapenem 10 ug-(CT1761B), Fusidic acid 10ug-(CT0023B), Gentamicin 10ug-(CT0024B), Imipenem 10ug-(CT0455B), Levofloxacin 5 ug- (CT1587B*)*, Lincomycin 15 ug-(CT0028B), Linezolid 30ug-(CT1650B), Meropenem 10ug- (CT0774B), Minocycline 30ug- (CT0030B), Moxifloxacin 1 ug-(CT1683B), Ofloxacin 5 ug –(CT0446B), Rifampicin 5 ug- (CT0207B), Tazobactam/piperacillin 105ug- (CT0725B), Teicoplanin 30 ug –(CT0647B), Tigecycline 15 ug-(CT1841B), Sulfamethoxazole/trimethoprim 25 ug – (CT0052B),Vancomycin 30ug-CT0058B)

### Isolation and identification of isolates

The isolates used in the current study were methicillin-resistant *S. aureus* isolates provided from private laboratories all over Alexandria, Egypt (25 isolates) as Swabs from conjunctivitis exudates the swabs were cultured on blood agar and mannitol salt agar for confirmation, catalase, coagulase, and hemolysin tests were performed as follows:

#### Hemolysin production

Isolates were streaked on 5–10% defibrinated human blood agar and incubated for 24 h at 37 ◦C, the clear zone around the streaks indicated β-hemolysis (hemolysin production) (Gerhard et al., 1994)^51^.

#### **Catalase activit**y

Catalase production was affirmed by the effervescence made upon the addition of H_2_O_2_ (3%) to a bacterial colony spread on a slide^52^.

#### Slide coagulase test

One drop of the bacterial suspension was mixed with one drop of citrated rabbit plasma on a cleaned glass slide. The slide was gently rocked for 5 to 10 s and examined for the presence of clumping^53^.

### **Antimicrobial susceptibility testing of the selected isolates.**

The Kirby Bauer technique was performed using different antibiotics discs against the selected isolates including oxacillin and cefoxitin (only isolates resistant to both were chosen as a phenotypic indication of Methicillin resistance): Amikacin, Amoxiclav, Ampicillin, Ampicillin/sulbactam, Azithromycin, Cefadroxil, Cefepime, Cefoperazone, Cefoperazone/sulbactam, Ceftazidime, Ceftriaxone, Cefuroxime, Ciprofloxacin, Clindamycin, Doxycycline, Ertapenem, Fusidic acid, Gentamicin, Imipenem, Levofloxacin, Lincomycin, Linezolid, Meropenem, Minocycline, Moxifloxacin, Ofloxacin, Rifampicin, Tazobactam/piperacillin, Teicoplanin, Tigecycline, Sulfamethoxazole/trimethoprim, Vancomycin^54^.

### Determination of biofilm-forming capacity of all isolates^55^

An assay of the biofilm-forming capacity of all isolates was done using a modified microtiter plate technique in the case of *S. aureus*. The overnight culture was adjusted to turbidity corresponding to 0.5 MacFarland (10^8^ colony forming units per milliliter, CFU/ml); then diluted to 10^6^ corresponding to 0.2 Macfarland, afterward, 200 µl of each dilution were aseptically transferred to wells of a flat bottom microtiter plate with lid, then incubated at 37 for 24 h, sterile Tryptic Soy Broth without bacterial inoculation was used as negative control, each isolate was done in triplicate, the content of the wells aspired and wells washed three times with 200 µl of sterile normal saline, then 200 µl of absolute methanol were applied for 15 min for fixation of formed biofilm if any.

Following, the plates were emptied and left to air dry, then 200 µl of 2% crystal violet was added to each well for 5 min then plates were emptied, washed, and air dried, and 160 µl 33% glacial acetic acid was used as eluent to the fixed crystal violet was added to each well before reading using ELISA reader at 630 nm.

According to the following criteria.

Non-biofilm producer$\:\:OD\le\:ODc$.

Weak biofilm producer<≤2×.

Moderate biofilm producer2×<≤4×.

Strong biofilm producer4×<.

### Detection of antimicrobial activity of Ibuprofen and its effect on levofloxacin activity^56^

The stock solution of levofloxacin was prepared by dissolving 400 mg of levofloxacin in 100 ml of sterile distilled water concentrations was calculated using CLSI M07 2012 Group U (for Quinolones Use) and 4 fold of the concentration were used considering the following checkerboard arrangement (*CLSI. Methods for Dilution Antimicrobial Susceptibility Tests for Bacteria That Grow Aerobically. 12th ed. CLSI standard M07.Clinical and Laboratory Standards Institute; 2024.)*

Ibuprofen was prepared to obtain 800 mg/ml stock solution in DMSO. The experiment was done in triplicate as follows: Sterile micro-titer plates were used, the horizontal wells were labeled 1,2,3…12; while vertical ones were labeled A, B, C. G. Part one: the wells A2 to A12 received 100 ul of two-fold serial dilution of the stock solution under test and 100 µl of double strength broth inoculated with organism under test at 10^6^ CFU/ml to determine MIC. Part two: The wells A2 to A12 received 50 µl of two-fold serially diluted stock solution of levofloxacin and 50 µl of sterile distilled water; while well A1 received 100 µl of sterile distilled water, which served as a control. Final concentrations of antibiotics under test ranged from 1000 to 0.9 µg/ml. The wells B1 to B12 received 50 µl of a two-fold serially diluted solution of levofloxacin and 50 µl of ibuprofen solution. The remaining series received the same two-fold serially diluted stock solution of the antimicrobial agent under test, and for series C, D, E, F, and G, respectively. Each of the 84 wells received 100 µl of sterile double-strength nutrient broth inoculated with 10^6^ CFU/ml of each of the tested organisms and mixed gently. The plates were covered and incubated at 35–37 °C for 14 h. The MIC was taken as the lowest antimicrobial agent concentration showing no growth turbidity. The combined activity of Levofloxacin and anti-inflammatories was calculated using the activity index method^56^ as follows:

$${\rm Activity \:Index =\frac{MIC\: of\: the\: antimicrobial \:agent \:in \:combination}{MIC \:of \:the \:antimicrobial \:agent \:alone}}$$

MIC of the antimicrobial agent alone.

### Determination of the antibiofilm activity of ibuprofen alone and in combination with levofloxacin antibiotic^56^

The same checkerboard arrangement mentioned above was used after 24 h, plates were emptied, washed 3 times with saline, and fixed with 200 µl of 99%methanol for 15 min, wells were decanted air dried, then the formed biofilms were dried by adding 200 µl 2% crystal violet for 5 min. Each well was eluted before reading by 160 µl 33% glacial acetic acid and read using an ELISA reader at 630 nm. All the experiments were performed in triplicate.

### MIC of the 3 formulations^56^

The three eye drop formulations were tested for their antimicrobial activity. The concentrations used were chosen according to the in-market available concentration and their two-fold serial dilutions in flat-bottom microtiter plates. The MIC was taken as the last well showing no turbidity.

### Antibiofilm activity of the 3 formulations^57^

The same arrangement mentioned in 2. 6 was used, after 24 h plates were emptied, washed 3 times with saline and fixed with 200 µl of 99% methanol for 15 min, wells were decanted, air dried, and then the formed biofilms were dried by adding 200 µl 2% crystal violet for 5 min. Each well was eluted before reading by 160 µl 33% glacial acetic acid and read using an ELISA reader at 630 nm. All the experiments were performed in triplicate.

### Determination of the rate of bactericidal activity of ibuprofen/levofloxacin combination using time-kill viable count technique

Systems were composed as follows: a mixture of 5 ml of 0.3% ibuprofen and 5 ml of 0.5% levofloxacin solution, a mixture of 5 ml of 0.3% ibuprofen and 5 ml of sterile normal saline, and a mixture of 5 ml 0.5% levofloxacin solution and 5 ml of sterile normal saline to mimic the concentration used in rat experiments. Aliquots of 10 ml sterile double-strength nutrient broth inoculated with 10^5^ CFU/ml of *S. aureus* chosen isolate (Panton valentine leucocidin toxin, PVL/mec A) were added to each of the two mixtures, mixed well, and placed at room temperature^58^. At time intervals 5,10,15,20,25,30 min, each reaction mixture was shaken well, and 100 µL aliquots were aseptically withdrawn and 10-fold serially diluted in sterile saline. The number of surviving organisms was determined by transferring 10 ml portions of each dilution into a 0.45 μm membrane filter placed in a sterilized filtration unit, then carried out under vacuum, and each membrane was washed 3 times with 100 ml sterile normal saline. The washed membranes were carefully removed from the filtration unit and placed with the upper surface up over the surface of nutrient agar plates, avoiding encountering air bubbles between the membrane and medium. The plates were then incubated at 37 °C for 48 h and the developing colonies were counted. Membranes showing between 30 and 300 CFU were used in the results. Concurrent with the experiment described above, A negative control with only Nutrient broth and a positive control with sterile double-strength nutrient broth inoculated with 10^5^ CFU/ml of *S. aureus* chosen isolate. All the experiments were performed in triplicate.

### Molecular detection of the virulence genes under test by polymerase chain reaction (PCR)

Fresh cultures of the selected *S.aureus* isolates were prepared on blood agar plates. Bacterial DNA was extracted by the boiling method^59^. The extracted DNA was evaluated by Nanodrop (Thomas Scientific, US)^60^. Each PCR reaction was performed in a reaction tube containing a 20 µl volume of 10 µl 2X PCR master mix, 0.5 µl of 10 picomoles of each primer, and 2 µl DNA extract. and remaining volume of PCR-grade water (according to the number of primers used). PCR amplification of the extracted DNA was carried out on the Veriti Thermal Cycler (Applied Biosystems, CA, USA). All primers used in this study and the annealing temperatures are listed in Table 2. The agarose at a concentration of 1–2%, according to separation, the type of ladder, and the expected size of the product, was prepared in TAE buffer for detecting the amplified PCR product^61^. PCR products were stained with 2 µg/ml ethidium bromide and were visualized under ultraviolet light.

**Table 2:** Primer sequences of studied bacterial genes.

Target gene	Primer	Band size	Annealing temperature
scpA^1^-F	AGGAGTTTTTATATGAAAAG	1200	54
scpA-R	TACCTTTCTAAAATACAAAT		
fnbB-F	GTAACAGCTAATGGTCGAATTGATACT	523	52
fnbB-R	CAAGTTCGATAGGAGTACTATGTTC		
*Hlb-F*	GTGCACTTACTGACAATAGTGC	309	55
*Hlb-R*	GTTGATGAGTAGCTACCTTCAGT		
*Hlg-F*	GTCAAAGAGTCCATAATGCATTTAA	535	55
*Hlg-R*	CACCAAATGTATAGCCTAAAGTG		
LUKDE^2^-F	TGAAAAAGGTTCAAAGTTGATACGAG	269	55
LUKDE-R	TGTATTCGATAGCAAAAGCAGTGCA		
mecA^3^	TCC AGA TTA CAA CTT CAC CAG G	162	55
	CCA CTT CAT ATC TTG TAA CG		
pvl	ATCAATAGGTAAAATGTCTGGACATGATCCA	433	55
	GCATCAAATGTATTGGATAG AAAAGC		
sea	GGTTATCAATGTGCGGGTGG	102	57
	CGGCACTTTTTTCTCTTCGG		
Eta^4^	GCAGGTGTTGATTTAGCATT	93	57
	AGATGTCCCTATTTTTGCTG		
tsst	ACCCCTGTTCCCTTATCATC	326	57
	TTTTCAGTATTTGTAACGCC		
scpA^3^-F	AGGAGTTTTTATATGAAAAG	1200	54
scpA-R	TACCTTTCTAAAATACAAAT		
fnbB-F	GTAACAGCTAATGGTCGAATTGATACT	523	52
fnbB-R	CAAGTTCGATAGGAGTACTATGTTC		
*Hlb* ^*5*^ *-F*	GTGCACTTACTGACAATAGTGC	309	55
*Hlb-R*	GTTGATGAGTAGCTACCTTCAGT		
*Hlg* ^*6*^*-F*	GTCAAAGAGTCCATAATGCATTTAA	535	55
*Hlg-R*	CACCAAATGTATAGCCTAAAGTG		
LUKDE-F	TGAAAAAGGTTCAAAGTTGATACGAG	269	55
LUKDE-R	TGTATTCGATAGCAAAAGCAGTGCA		

scpA^1^, staphopain A.

LUKDE^2^, leucocidin genes.

MecA^3^, one of the genes coding for methicillin resistance.

Eta^4^ gene, encodes a member of the Y family of specialized DNA polymerases. It copies undamaged DNA with a lower fidelity.

; Hlb^5^ and Hlg^6^, Hemolysin genes.

### Molecular docking of ibuprofen using the Mcule online website

To preliminarily elucidate the mechanism of action of ibuprofen, a survey on different targets in *S. aureus* was done using the Mcule library online.

### Experimental animals

At the time of the test, 15 male Sprague-Dawley rats weighing 170–200 g and 7–9 weeks of age were obtained from the animal facility unit, Faculty of Medicine, Alexandria University, Egypt. Five rats per cage were accommodated in animal cages at 23 °C in a 12 h light/12 h dark cycle, with free water and a standard chow diet, for 1 week before the experiment. The study is reported in accordance with ARRIVE guidelines. All procedures were performed per the Institutional Animal Care and Use Committee (IACUC), Alexandria University, Faculty of Medicine, Egypt (Approval number: AU012234931), and fulfilled the “National Research Council’s guide for the care and use of laboratory animals”^62^. Every attempt was made to restrict the struggle of the rats throughout the experiment.

### Experimental design

Keratitis was induced in male Sprague Dawley rats, where each rat was anesthetized by intraperitoneal injection of 40 mg/kg pentobarbital sodium. One drop of 0.5% proparacaine was applied as a topical anesthesia in the right eye, which was wounded with abrasions using a 26-gauge needle that only penetrated the epithelial cell layer of the cornea^63^. The injured-induced rats were randomly assigned into five experimental groups using a computer-generated randomization method (*n* = 3 per group): group I is the control rats, the non-infected corneal abrasion group without treatments, group II; infected-keratitis (IK) rats (*S. aureus*, without treatment), group III; levofloxacin (IK rats treated with 0.5% levofloxacin eye drops), group IV; ibuprofen (IK rats treated with 0.3% ibuprofen eye drops), and group V; levofloxacin+ibuprofen (IK rats treated with combination therapy of 0.5% levofloxacin/0.3% ibuprofen eye drops). Bacterial keratitis was induced by topical inoculation of a 10 µl aliquot containing *S. aureus* (one chosen to isolate harboring both PVL and mecA genes) suspension containing approximately 1 × 10⁶ CFU/mL to the scratched cornea of the rats. Infected keratitis (IK) rats were examined to ensure the presence of an inflamed cornea characterized by dense opacity^64^. The control rats were administered plain normal saline without drugs. After two weeks of treatments, rats were killed by cervical dislocation, and the eyes were divided into two aliquots. The first aliquot was stored at −80 °C for reverse transcription-polymerase chain reaction (RT-PCR) analysis, and the other part of the eye was fixed in 10% formalin for histological examination. The molecular and histological analyses performed blinded to treatment groups.

### Reverse transcription-polymerase chain reaction (RT-PCR) analysis

All samples were analyzed in triplicate. GAPDH was used as an internal reference gene for normalization. Relative gene expression was calculated using the ΔΔCt method. Primer specificity was verified, and amplification efficiency was within an acceptable range (90–110%). Total RNA was isolated from corneal tissue using the RNeasy kit and according to the manufacturer’s instructions. The isolated RNA was reverse transcribed into complementary DNA (cDNA) using reverse transcriptase, amplified, and detected by qRT-PCR using specific primers. Primer sequences of the studied genes are provided in Table 2. Reverse transcription RNA to cDNA was performed using QuantiTect Reverse Transcription Kit (Catalog no. 20531, Qiagen, Germany) and according to the manufacturer’s instructions. The cDNA was used to quantify the corneal expression of TNF-α, VEGF-A, IL-1β, IL*-*17 A, TLR4, apoptosis regulator BCL2-associated X (Bax), MMP2, and MMP9 genes by Rotor-Gene Q qPCR using QuantiTect SYBR Green PCR Master Mix using specific primer sets for each gene as presented in Table 3. Quantitative PCR assay was carried out under the following conditions: initial denaturation for 10 min at 95 ◦C and then amplification by 40 cycles of PCR: denaturation at 95 ◦C for 15 s, annealing at 58 ◦C for 15 s, and extension at 60 ◦C for 15 s. Values of the threshold cycle (Ct) were determined by Rotor-Gene Q-Pure Detection version 2.1.0 (build 9)^65^.

**Table 3:** Primer sequences of studied rat genes.

Gene name	Access No.	primer	Sequence
**VEGF-A**	**NM_031836.3**	Forward	CTGCTGTAACGATGAAGCCCTG
		Reverse	GCTGTAGGAAGCTCATCTCTCC
**TNF-α**	**NM_012675.3**	Forward	ACCACGCTCTTCTGTCTACTG
		Reverse	CTTGGTGGTTTGCTACGAC
**IL-1β**	**NM_031512.2**	Forward	TGGACCTTCCAGGATGAGGACA
		Reverse	GTTCATCTCGGAGCCTGTAGTG
**IL-17 A**	**NM_001106897.1**	Forward	CTTCACCCTGGACTCTGAGC
		Reverse	TGGCGGACAATAGAGGAAAC
**TLR4**	**NM_019178.2**	Forward	AGCTTTGGTCAGTTGGCTCT
		Reverse	CAGGATGACACCATTGAAGC
**Bax**	**NM_017059.2**	Forward	GCGAATTGGCGATGAACTG
		Reverse	ATGGTTCTGATCAGCTCGG
**MMP2**	**NM_031054**	Forward	ACCGTCGCCCATCATCAA
		Reverse	TTGCACTGCCAACTCTTTGTCT
**MMP9**	**NM_031055.2**	Forward	TCGAAGGCGACCTCAAGTG
		Reverse	TTCGGTGTAGCTTTGGATCCA
**GAPDH**	**NM_017008**	Forward	GGGTGTGAACCACGAGAAATA
		Reverse	AGTTGTCATGGATGACCTTGG

### Histological examination

Part of the corneas of the 15 rats (3 cornea samples per group) were fixed in 10% formalin for histological examination. After fixation, the cornea samples were embedded in paraffin using the conventional method. Fifteen corneal sections of 4 μm were serially prepared and HE-stained. Histological sections were evaluated by a blinded pathologist using standardized criteria, including epithelial integrity, inflammatory infiltration, and stromal architecture.

### Statistical analysis

Values were expressed as means ± SD. One-way analysis of variance (ANOVA) was used for statistical analysis of the results, followed by the Tukey-Kramer test to appropriately control for multiple group comparisons as the post hoc test. Statistical significance was defined as *p* < 0.05.

## Supplementary Information

Below is the link to the electronic supplementary material.

Supplementary Material 1

## Data Availability

“The dataset(s) [66] supporting the conclusions of this article is(are) included within the article (and its additional file(s)).” https://doi.org/10.6084/m9.figshare.28102712.v1.

## Declarations

### Dual publication

The submitted work is original and has not been published elsewhere in any form or language.
