# Supplementary material for: An eye drop combination for treating Staphylococcus aureus-induced keratitis in rats: repurposing ibuprofen
Source: Sci Rep. 2026 Apr 24;16:13387. doi: 10.1038/s41598-026-48096-z (PMC13109396; doi:10.1038/s41598-026-48096-z)
Supplement: Supplementary file 1 — Supplementary Material 1 [file 41598_2026_48096_MOESM1_ESM.pdf]

Supplementary data

Expression of IL-17A (fold change)

ANOVA summary

F	3.491
P value	0.0495
P value summary	*
Significant diff. among means (P < 0.05)?	Yes
R squared	0.5827

Tukey's multiple comparisons test	Mean diff.	95.00% CI of diff.	Below threshold?	Summary
Control vs. IK	-0.4967	-1.436 to 0.4424	No	ns
Control vs. IK+LVX	-0.1367	-1.076 to 0.8024	No	ns
Control vs. IK+IBP	-0.9600	-1.899 to -0.02098	Yes	*
Control vs. IK+LVX+IBP	-0.5400	-1.479 to 0.3990	No	ns
IK vs. IK+LVX	0.3600	-0.5790 to 1.299	No	ns
IK vs. IK+IBP	-0.4633	-1.402 to 0.4757	No	ns
IK vs. IK+LVX+IBP	-0.04333	-0.9824 to 0.8957	No	ns
IK+LVX vs. IK+IBP	-0.8233	-1.762 to 0.1157	No	ns
IK+LVX vs. IK+LVX+IBP	-0.4033	-1.342 to 0.5357	No	ns
IK+IBP vs. IK+LVX+IBP	0.4200	-0.5190 to 1.359	No	ns

Expression of IL-1B (Fold change)

ANOVA summary

F	6.817
P value	0.0065
P value summary	**
Significant diff. among means (P < 0.05)?	Yes
R squared	0.7317

Tukey's multiple comparisons test	Mean diff.	95.00% CI of diff.	Below threshold?	Summary
Control vs. IK	-1.767	-2.965 to -0.5681	Yes	**
Control vs. IK+LVX	167	-2.015 to 0.3819	No	ns
Control vs. IK+IBP	-0.5667	-1.765 to 0.6319	No	ns
Control vs. IK+LVX+IBP	-0.3133	-1.512 to 0.8852	No	ns
IK vs. IK+LVX	0.9500	-0.2486 to 2.149	No	ns
IK vs. IK+IBP	1.200	0.001435 to 2.399	Yes	*
IK vs. IK+LVX+IBP	1.453	0.2548 to 2.652	Yes	*
IK+LVX vs. IK+IBP	0.2500	-0.9486 to 1.449	No	ns
IK+LVX vs. IK+LVX+IBP	0.5033	-0.6952 to 1.702	No	ns
IK+IBP vs. IK+LVX+IBP	0.2533	-0.9452 to 1.452	No	ns

Expression of TNF-a(Fold change)

ANOVA summary	
F	29.38
P value	<0.0001
P value summary	****
Significant diff. among means (P < 0.05)?	Yes
R squared	0.9216

Tukey's multiple comparisons test	ean diff.	95.00% CI of diff.	Below threshold?	Summary
Control vs. IK	-5.600	-7.579 to -3.621	Yes	****
Control vs. IK+LVX	-1.027	-3.006 to 0.9528	No	ns
Control vs. IK+IBP	-0.8667	-2.846 to 1.113	No	ns
Control vs. IK+LVX+IBP	-0.2433	-2.223 to 1.736	No	ns
IK vs. IK+LVX	4.573	2.594 to 6.553	Yes	***
IK vs. IK+IBP	4.733	2.754 to 6.713	Yes	***
IK vs. IK+LVX+IBP	5.357	3.377 to 7.336	Yes	****
IK+LVX vs. IK+IBP	0.1600	-1.819 to 2.139	No	ns
IK+LVX vs. IK+LVX+IBP	0.7833	-1.196 to 2.763	No	ns
IK+IBP vs. IK+LVX+IBP	0.6233	-1.356 to 2.603	No	ns

Expression of TLR4 (Fold change)

ANOVA summary

F	6.977
P value	0.0060
P value summary	**
Significant diff. among means (P < 0.05)?	Yes
R squared	0.7362

Tukey's multiple comparisons test	Mean diff.	95.00% CI of diff.	Below threshold?	Summary
Control vs. IK	-3.610	-6.238 to -0.9817	Yes	**
Control vs. IK+LVX	-3.340	-5.968 to -0.7117	Yes	*
Control vs. IK+IBP	-1.787	-4.415 to 0.8416	No	ns
Control vs. IK+LVX+IBP	-1.317	-3.945 to 1.312	No	ns
IK vs. IK+LVX	0.2700	-2.358 to 2.898	No	ns
IK vs. IK+IBP	1.823	-0.8050 to 4.452	No	ns
IK vs. IK+LVX+IBP	2.293	-0.3350 to 4.922	No	ns
IK+LVX vs. IK+IBP	1.553	-1.075 to 4.182	No	ns
IK+LVX vs. IK+LVX+IBP	2.023	-0.6050 to 4.652	No	ns
IK+IBP vs. IK+LVX+IBP	0.4700	-2.158 to 3.098	No	ns

Expression of MMP2 (Fold change)

ANOVA summary	
F	5.058
P value	0.0172
P value summary	*
Significant diff. among means (P < 0.05)?	Yes
R squared	0.6692

Tukey's multiple comparisons test	Mean diff.	95.00% CI of diff.	Below threshold?	Summary
Keratitis vs. IK	-2.117	-3.900 to -0.3329	Yes	*
Keratitis vs. IK+LVX	-1.310	-3.094 to 0.4738	No	ns
Keratitis vs. IK+IBP	-0.5867	-2.370 to 1.197	No	ns
Keratitis vs. IK+LVX+IBP	-0.2467	-2.030 to 1.537	No	ns
IK vs. IK+LVX	0.8067	-0.9771 to 2.590	No	ns
IK vs. IK+IBP	1.530	-0.2538 to 3.314	No	ns
IK vs. IK+LVX+IBP	1.870	0.08621 to 3.654	Yes	*
IK+LVX vs. IK+IBP	0.7233	-1.060 to 2.507	No	ns
IK+LVX vs. IK+LVX+IBP	1.063	-0.7205 to 2.847	No	ns
IK+IBP vs. IK+LVX+IBP	0.3400	-1.444 to 2.124	No	ns

Expression of MMP9 (Fold change)

ANOVA summary

F	13.29
P value	0.0005
P value summary	***
Significant diff. among means (P < 0.05)?	Yes
R squared	0.8416

Tukey's multiple comparisons test	Mean diff.	95.00% CI of diff.	Below threshold?	Summary
Keratitis vs. IK	-1.080	-1.718 to -0.4423	Yes	**
Keratitis vs. IK+LVX	-0.9500	-1.588 to -0.3123	Yes	**
Keratitis vs. IK+IBP	-0.3433	-0.9810 to 0.2943	No	ns
Keratitis vs. IK+LVX+IBP	-0.07000	-0.7077 to 0.5677	No	ns
IK vs. IK+LVX	0.1300	-0.5077 to 0.7677	No	ns
IK vs. IK+IBP	0.7367	0.09899 to 1.374	Yes	*
IK vs. IK+LVX+IBP	1.010	0.3723 to 1.648	Yes	**
IK+LVX vs. IK+IBP	0.6067	-0.03101 to 1.244	No	ns
IK+LVX vs. IK+LVX+IBP	0.8800	0.2423 to 1.518	Yes	**
IK+IBP vs. IK+LVX+IBP	0.2733	-0.3643 to 0.9110	No	ns

VEGF-A expression (fold change)

ANOVA summary

F	5.847
P value	0.0108
P value summary	*
Significant diff. among means (P < 0.05)?	Yes
R squared	0.7005

Tukey's multiple comparisons test	Mean diff.	95.00% CI of diff.	Below threshold?	Summary
Control vs. IK	-2.660	-4.864 to -0.4564	Yes	*
Control vs. IK+LVX	-2.330	-4.534 to -0.1264	Yes	*
Control vs. IK+IBP	-0.9033	-3.107 to 1.300	No	ns
Control vs. IK+LVX+IBP	-0.6033	-2.807 to 1.600	No	ns
IK vs. IK+LVX	0.3300	-1.874 to 2.534	No	ns
IK vs. IK+IBP	1.757	-0.4470 to 3.960	No	ns
IK vs. IK+LVX+IBP	2.057	-0.1470 to 4.260	No	ns
IK+LVX vs. IK+IBP	1.427	-0.7770 to 3.630	No	ns
IK+LVX vs. IK+LVX+IBP	1.727	-0.4770 to 3.930	No	ns
IK+IBP vs. IK+LVX+IBP	0.3000	-1.904 to 2.504	No	ns

Expression of Bax (Fold change)

ANOVA summary

F	37.27
P value	<0.0001
P value summary	****
Significant diff. among means (P < 0.05)?	Yes
R squared	0.9371

Tukey's multiple comparisons test	Mean diff.	95.00% CI of diff.	Below threshold?	Summary
Control vs. IK	-6.587	-8.701 to -4.473	Yes	****
Control vs. IK+LVX	-5.297	-7.411 to -3.183	Yes	****
Control vs. IK+IBP	-1.930	-4.044 to 0.1842	No	ns
Control vs. IK+LVX+IBP	-1.423	-3.537 to 0.6908	No	ns
IK vs. IK+LVX	1.290	-0.8242 to 3.404	No	ns
IK vs. IK+IBP	4.657	2.543 to 6.771	Yes	***
IK vs. IK+LVX+IBP	5.163	3.049 to 7.277	Yes	****
IK+LVX vs. IK+IBP	3.367	1.253 to 5.481	Yes	**
IK+LVX vs. IK+LVX+IBP	3.873	1.759 to 5.987	Yes	***
IK+IBP vs. IK+LVX+IBP	0.5067	-1.607 to 2.621	No	ns
